# Sex differences in hospitalisation and healthcare utilisation for patients with atrial fibrillation *Middeldorp et al. Sex differences in healthcare utilisation and AF*

**DOI:** 10.1016/j.ijcha.2025.101748

**Published:** 2025-07-12

**Authors:** Melissa E. Middeldorp, Colinda van Deutekom, Liann I. Weil, Isabelle C. Van Gelder, Ursula W. De Ruijter, Patrick T. Jeurissen, Emelia J. Benjamin, Barbara C. van Munster, Michiel Rienstra

**Affiliations:** a Department of Cardiology, University of Groningen, University Medical Center Groningen, Groningen, the Netherlands; b University of Groningen, University Medical Center Groningen, Department of Geriatric Medicine, Groningen, the Netherlands; c Erasmus University Medical Center, Department of Public Health, Rotterdam, the Netherlands; d Radboud University Medical Center, Radboud Institute for Health Sciences, Scientific Center for Quality of Healthcare (IQ Healthcare), Nijmegen, the Netherlands; e Department of Medicine, Boston Medical Center, Boston University Chobanian & Avedisian School of Medicine, United States of America; f Department of Epidemiology, Boston University School of Public Health, United States of America

**Keywords:** Atrial fibrillation, Sex, Healthcare utilisation, Age

## Abstract

**Background:**

There is limited data on sex differences in healthcare utilization among patients with atrial fibrillation (AF). This study aimed to assess the association of sex and age on healthcare utilization in AF patients.

**Methods:**

We conducted a retrospective analysis of electronic health records from three hospitals in the Netherlands, including all patients ≥ 18 years with at least one healthcare encounter (outpatient, emergency visit, or inpatient stay). AF diagnoses were identified using ICD-10 codes linked with the Dutch Hospital Data Clinical Classification Software.

**Results:**

Of 226,991 patients, 5127 (2.3 %) had AF (44 % females, mean age 68 ± 12 years). There were no sex differences in outpatient, emergency, or inpatient visits overall. However, females aged 18–59 had more outpatient visits compared to males (6.1 ± 7.9 vs 4.8 ± 5.2, p = 0.001). In contrast, females aged ≥ 75 had fewer outpatient visits (7.2 vs 8.4, p < 0.001) and inpatient days (4.8 vs 5.8, p = 0.027) compared to males. After multivariable adjustment, both sexes aged ≥ 75 had increased risks of inpatient stays (Females: OR 2.53, 95 % CI 2.30–2.78; Males: OR 1.49, 95 % CI 1.46–1.62) and emergency visits (Females: OR 2.14, 95 % CI 1.94–2.35; Males: OR 1.13, 95 % CI 1.03–1.24). Significant interactions between sex and age were found, with females having higher odds of inpatient days (OR 1.99, p < 0.001) and emergency visits (OR 1.23, p < 0.001) compared to males.

**Conclusion:**

While no overall sex differences in healthcare utilization were found, significant age-related differences were observed, with females having higher hospital utilization rates, particularly for inpatient stays and emergency visits.

## Graphical abstract

**Figure:**
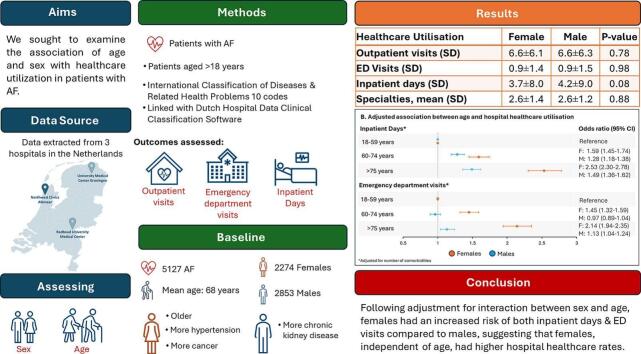

## 1 Introduction

Despite Atrial Fibrillation (AF) being the most common sustained cardiac arrhythmia, [1] the understanding of sex differences in AF presentation, management, and outcomes remains incomplete [2]. Emerging evidence suggests that there are differences in management of men and women with AF which may influence their health care utilization patterns [[3], [4], [5]]. Females with AF often present with more severe symptoms [6] yet studies have shown they are less likely to receive optimal care compared to males [5]. Inequities may be attributable to biological differences, such as hormonal influences and comorbid conditions [3,7].

Females with AF tend to have higher rates of comorbidities compared to males, which may influence their health care utilization patterns [[8], [9], [10], [11]]. Data from the Rate Control Efficacy in Permanent Atrial Fibrillation (RACE II) study in females with permanent AF demonstrated females had a greater accumulation of risk factors compared to males, which negatively impacted their quality of life [11]. The presence of multiple chronic conditions can lead to increased hospital admissions, more frequent outpatient visits, and a greater need for diagnostic and therapeutic interventions [12,13]. Understanding the interplay between sex and health care utilization is crucial for developing comprehensive management strategies for AF patients and inform the development of targeted strategies to improve outcomes and resource allocation [14].

This study aims to examine sex differences amongst patients with AF in health care utilization, specifically outpatient visits, emergency department visits and inpatients days as well as the specialists involved in the management and along with the influence of age in this population.

## 2 Methods

The present study is a retrospective analysis of administrative electronic health records from two academic medical centres and one general hospital in the Netherlands. The two academic centres included were the University Medical Center Groningen (UMCG) and Radboud University Medical Center (Radboud UMC) and the general hospital included was Northwest Clinics Alkmaar (NWCA).

### 2.1 Data extraction

Data extraction was undertaken utilising electronic health records data, which were stored for administrative and billing purposes. We utilised data from the UMCG and Radboud UMC (January 1, 2017 – December 31, 2018) and from NWCA (January 1 – December 31, 2019). For patients from UMCG and Radboud UMC we included data from their initial presentation year only, and the subsequent year admission for these individuals were excluded to avoid duplication. Healthcare utilisation type was determined based on Diagnosis-Treatment Combinations, this is the coding for hospital care and claiming payments commonly used in the Netherlands [15]. Data was obtained from each hospital including age, sex, inpatient days, emergency department visits, and outpatient visits per patient. Additionally, we collected the type and number of diagnoses for each outpatient visit per patient and the number and type of medical specialists involved per outpatient visit. We included all patients aged 18 years or older who had at least one healthcare utilisation with one of the three hospitals. Deidentified patient data were provided and patients who declined the use of their data were excluded prior to data collection.

The Central Ethics Review Board of the UMCG approved the pseudonymous use of data for research purposes for all three hospitals. The approval was recognised by the Radboud UMC and NWCA for the use of their data. Data transfer agreements were signed between the UMCG, the Radboud UMC and NWCA.

### 2.2 Diagnosis codes

Diagnosis of patients treated at each hospital were determined using the International Classification of Diseases and Related Health Problems 10 (ICD-10) codes, which were registered within the Diagnosis-Treatment Combinations; ICD codes were linked with the Dutch Hospital Data – Clinical Classification Software (DHD-CCS) [16]. In order to define AF, we utilised the DHD-CCS coding for atrial arrhythmias including those with a primary diagnosis of AF. To determine multimorbidity in patients the number of comorbidities were defined based on the number of diagnoses as deemed by the DHD-CCS classification code *(See* supplemental file*).*

### 2.3 Statistical analysis

Patient characteristics of those with AF were reported stratified by sexes. Variables were presented as means (standard deviation (SD)) for continuous variables when normally distributed and as median (25^th^ and 75^th^ percentile) when not normally distributed, for categorical variables we reported frequencies (percentages). The groups were compared using *t* test or Kruskal-Wallis test (if normality assumption was violated) for continuous variables and χ^2^ Fisher’s Exact test for categorical variables. To determine association of age with healthcare utilisation, stratified analysis was also undertaken in the following 3 categories: 18–59 years, 60–74 years and ≥ 75 years. Risk of inpatient visit and emergency department visit was assessed using both logistic and linear regression models in females and males separately. Logistic regression was used to estimate the likelihood of binary outcomes of a presentation, and results were expressed as odds ratios with 95 % confidence intervals. Linear regression was applied for continuous outcomes, and results were reported as regression coefficients with 95 % confidence intervals. Both models were initially unadjusted and subsequently adjusted for key covariates, including age and number of comorbidities, to account for potential confounding. To assess the interaction between sex and age, we also undertook further logistic regression including an interaction term (sex x age) in the model.

Data processing was performed using R (Version 4.3.3, R Foundation for Statistical Computing, Vienna, Austria). Analyses were performed with STATA software (version 18.0, StataCorp LLC, Texas, USA). A p-value < 0.05 was considered statistically significant.

## 3 Results

There was a total of 226,991 patients, 5127 (2.3 %) diagnosed with AF: 1448 (28 %) from the UMCG, 2152 (42 %) from Radboud UMC and 1527 (30 %) from NWCA. Of these 2274 (44 %) were women with a mean overall age of the patients was 68 ± 12 years. Female patients with AF were older (mean age: 69 ± 13 vs 67 ± 12 years) and had more cancer (27 % vs 21 %) compared to males (Table 1).

**Table 1:** Baseline characteristics of males and females with AF.

	**AF**		**p-value**
	**Female**	**Male**	
**N=**	2,274	2,853	
Age, mean (SD)	68.6 ± 12.6	67.4 ± 12.0	0.001
Number of comorbidities, mean (SD)	2.9 ± 1.9	2.9 ± 1.9	0.98
Acute cerebrovascular disease (%)	38 (2)	62 (2)	0.19
Cancer (%)	602 (27)	601 (21)	<0.001
Chronic kidney disease (%)	56 (3)	98 (3)	0.043
Chronic obstructive pulmonary disease (%)	71 (3)	112 (4)	0.12
Diabetes (%)	46 (2)	72 (3)	0.24
Hypertension (%)	48 (2)	36 (1)	0.017
Heart Failure (%)	106 (5)	116 (4)	0.30
Valvular disease (%)	28 (1)	35 (1)	0.98
Myocardial infarction (%)	21 (1)	17 (1)	0.17
Other cerebrovascular disease (%)	4 (0.2)	5 (0.2)	0.99
Syncope (%)	11 (1)	15 (1)	0.83

### 3.1 Healthcare by sex

When comparing females to males with AF, there was no difference in the median number of outpatient visits (6.6 ± 6.1 vs 6.6 ± 6.3) or emergency department visits (0.9 ± 1.4 vs 0.9 ± 1.5). There was a trend to less inpatient days (3.7 ± 8.0 vs 4.2 ± 9.0, p = 0.08). Similarly, there was no difference between females or males with AF in the mean number of medical specialists seen (2.6 ± 1.4 vs 2.6 ± 1.2) (Table 2).

**Table 2:** Healthcare utilization comparing males and females with AF.

	**AF**		**p-value**
	**Female**	**Male**	
**N=**	2,274	2,853	
Outpatient visits, median (Q1, Q3)	5 (2, 9)	5 (2, 9)	0.78
ED visits, median (Q1, Q3)	0 (0, 1)	0 (0, 1)	0.98
Inpatient days, median (Q1, Q3)	0 (0, 4)	0 (0, 4)	0.08

**Number of medical specialties**			
1	664 (29)	843 (30)	
2	522 (23)	662 (23)	
3	449 (20)	583 (20)	0.90
4	304 (13)	388 (14)	
5+	305 (13)	377 (13)	
Involved specialties, mean (SD)	2.6 ± 1.4	2.6 ± 1.2	0.88

**Medical Specialist**			
Anaesthesiology (%)	66 (3)	55 (2)	0.022
Cardiology (%)	1805 (79)	2622 (92)	<0.001
Cardiothoracic surgery (%)	23 (1)	64 (2)	0.001
Clinical genetics (%)	22 (1)	19 (0.7)	0.17
Dermatology (%)	216 (10)	286 (10)	0.53
Gastroenterology (%)	255 (11)	251 (9)	0.004
General surgery (%)	355 (16)	441 (16)	0.88
Geriatrics (%)	93 (4)	105 (4)	0.45
Gynaecology (%)	220 (10)	1 (0.04)	<0.001
Internal medicine (%)	667 (29)	810 (28)	0.46
Neurology (%)	349 (15)	482 (17)	0.14
Neurosurgery (%)	45 (2)	44 (2)	0.23
Ophthalmology (%)	211 (9)	251 (9)	0.55
Orthopaedic surgery (%)	224 (10)	208 (7)	0.001
Otorhinolaryngology (%)	160 (7)	222 (8)	0.31
Physiatry Rehabilitation (%)	12 (1)	29 (1)	0.05
Plastic surgery (%)	50 (2)	55 (2)	0.50
Psychiatry (%)	2 (0.1)	5 (0.2)	0.44
Pulmonology (%)	297 (13)	443 (16)	0.013
Radiotherapy (%)	319 (14)	114 (4)	<0.001
Rheumatology (%)	172 (18)	156 (6)	0.002
Urology (%)	97 (4)	378 (13)	<0.001

ED: Emergency department.

The type of specialist seen by both sexes with AF are shown in Table 2. Females saw more gastroenterology, gynaecology, orthopaedic surgery, radiotherapy, and rheumatology specialists than males, and fewer cardiology, cardiothoracic surgery, and urology specialists than males.

### 3.2 Comorbidities in females and males by age

In females with AF the mean number of comorbidities was higher in higher age categories, ranging from 2.6 ± 1.7 for those 18–59 years to 3.3 ± 2.0 for those ≥ 75 years (p < 0.001) (Table 3). For males with AF there was a more pronounced increase with advancing age from 2.1 ± 1.5 for those 18–59 years to 3.6 ± 2.0 for those ≥ 75 years (p < 0.001). The most common comorbidities for females and males with AF and ≥ 75 years were cancer and heart failure.

**Table 3:** Comorbidities between sexes by age with AF.

**Years of age:**	**Female**					**Male**			
	**18**–**59**	**60**–**74**	≥**75**	**p-value**		**18**–**59**	**60**–**74**	≥**75**	**p-value**
**N=**	473	981	820			634	1,426	793	
Age (SD)	49.4 ± 8.7	67.8 ± 4.0	80.5 ± 4.5	<0.001		50.4 ± 8.9	67.8 ± 4.2	80.4 ± 4.5	<0.001
Number of comorbidities, (SD)	2.6 ± 1.7	2.8 ± 1.8	3.3 ± 2.0	<0.001		2.1 ± 1.5	2.8 ± 1.8	3.6 ± 2.0	<0.001
Prior cerebrovascular event*, n (%)	7 (2)	15 (2)	16 (2)	0.73		7 (1)	29 (2)	26 (3)	0.02
Cancer, n (%)	129 (27)	254 (36)	219 (27)	0.84		58 (9)	284 (20)	259 (33)	<0.001
Chronic kidney disease, n (%)	4 (1)	23 (2)	29 (4)	0.01		4 (1)	44 (3)	50 (6)	<0.001
COPD, n (%)	7 (2)	34 (4)	30 (4)	0.07		7 (1)	58 (4)	47 (6)	<0.001
Diabetes, n (%)	13 (3)	11 (1)	22 (3)	0.03		8 (1)	40 (3)	24 (3)	0.07
Hypertension, n (%)	7 (2)	21 (2)	20 (2)	0.51		6 (1)	15 (1)	15 (2)	0.17
Heart Failure, n (%)	8 (2)	32 (3)	66 (8)	<0.001		10 (2)	49 (3)	57 (7)	<0.001
Valvular disease, n (%)	4 (1)	8 9 (1)	16 (2)	0.07		6 (1)	12 (1)	17 (2)	0.02
Myocardial infarction, n (%)	0	9 (1)	12 (2)	0.03		3 (1)	7 (1)	7 (1)	0.47
Syncope, n (%)	1 (0.2)	6 (1)	4 (1)	0.59		4 (1)	6 (0.4)	5 (1)	0.74

* Cerebrovascular event was defined as intracerebral hematoma, chronic subdural hematoma/hygroma, cerebrovascular accident/Transient ischemic attack, or, intracranial, subarachnoidal or intracerebral hemorrhage.

### 3.3 Healthcare utilisation in females and males by age

There was a higher number of healthcare utilisation between female and male patients with AF when stratified by age (Table 4). The number of outpatient visits, emergency department visits, and inpatient days for female patients with AF was increased from those aged 18–59 up to those aged ≥ 75 years of age (all p < 0.001). In male patients with AF there was also a higher number of outpatient visits, emergency department visits and inpatient days in those aged ≥ 75 years of age compared to those aged 18–59 years of age (all p < 0.001).

**Table 4:** Healthcare utilization in females and males with AF by age group.

	**Female**					**Male**					**p-value between groups**		
	**18**–**59**	**60**–**74**	≥**75**	**p-value**		**18**–**59**	**60**–**74**	≥**75**	**p-value**		**18**–**59**	**60**–**74**	≥**75**
	473	981	820			634	1,426	793					
Outpatient visits, median (Q1, Q3)	4 (2,8)	5 (2,9)	6 (3,10)	<0.001		3 (2,6)	4 (2,8)	6 (4,11)	<0.001		0.002	0.832	0.001
ED visits, median (Q1, Q3)	0 (0,1)	0 (0,1)	1 (0,2)	<0.001		0 (0,1)	0 (0,1)	1 (0,2)	<0.001		0.006	0.486	0.739
Inpatient days, median (Q1, Q3)	0 (0,2)	0 (0,2)	0 (0,6)	<0.001		2 (1,3)	2 (1,3)	3 (2,4)	<0.001		0.886	0.101	0.027

**Number of medical specialties**													
1	166 (35.1)	333 (33.9)	165 (20.1)			299 (47)	434 (30)	110 (14)					
2	142 (30.0)	228 (23.2)	182 (22.2)			188 (30)	344 (24)	152 (19)					
3	77 (16.3)	184 (18.8)	188 (22.9)	<0.001		94 (15)	299 (21)	190 (24)	<0.001		0.001	0.418	0.001
4	45 (9.5)	123 (12.5)	136 (16.6)			39 (6)	180 (13)	169 (21)					
5+	43 (9.1)	113 (11.5)	149 (18.2)			36 (6)	169 (12)	172 (22)					
Involved medical specialties, mean (SD)	2.3 ± 1.5	2.5 ± 1.6	3.0 ± 1.7	<0.001		2.0 ± 1.3	2.6 ± 1.6	3.4 ± 1.7	<0.001		<0.001	0.221	<0.001

AF: atrial fibrillation; CVD: cardiovascular disease; ED: emergency department.

The UMCG, which employs MR, has received consultancy fees from Bayer and InCarda Therapeutics. All the other authors have nothing to disclose.

When comparing age between females to males, females had a significantly greater number of outpatient visits (6.1 vs 4.8, p = 0.001) in patients with AF aged 18–59 years, whilst males had a significantly higher number of emergency department visits 0.9 vs 0.7 (p = 0.006) compared to females (Table 4). There was no significant difference in inpatient days (3.1 vs 3.0, p = 0.886) for females and males with AF aged 18–59 years. When comparing female to male patients with AF aged > 75 years, there was significantly greater number of outpatient visits (7.2 vs 8.4, p < 0.001) and inpatient days (4.8 vs 5.8, p = 0.03), however there was no significant difference in emergency department visits (1.1 vs 1.1, p = 0.74).

The number of medical specialists also increased incrementally across age categories from 2.3 in females with AF aged 18–59 years up to 3.0 in females with AF aged ≥ 75 years (p < 0.001). In males with AF there was also an increase in the medical specialists from 2.0 in those aged 18–59 years up to 3.4 in those aged ≥ 75 years (p < 0.001). (Table 4) There were 35 % females with AF aged ≥ 75 years compared 43 % males with AF aged ≥ 75 years seeing ≥ 4 specialists (p < 0.001). The type of specialist seen by each age category is outlined in Supplemental table 2.

### 3.4 Association between sex and healthcare utilization

**Fig. 1:**
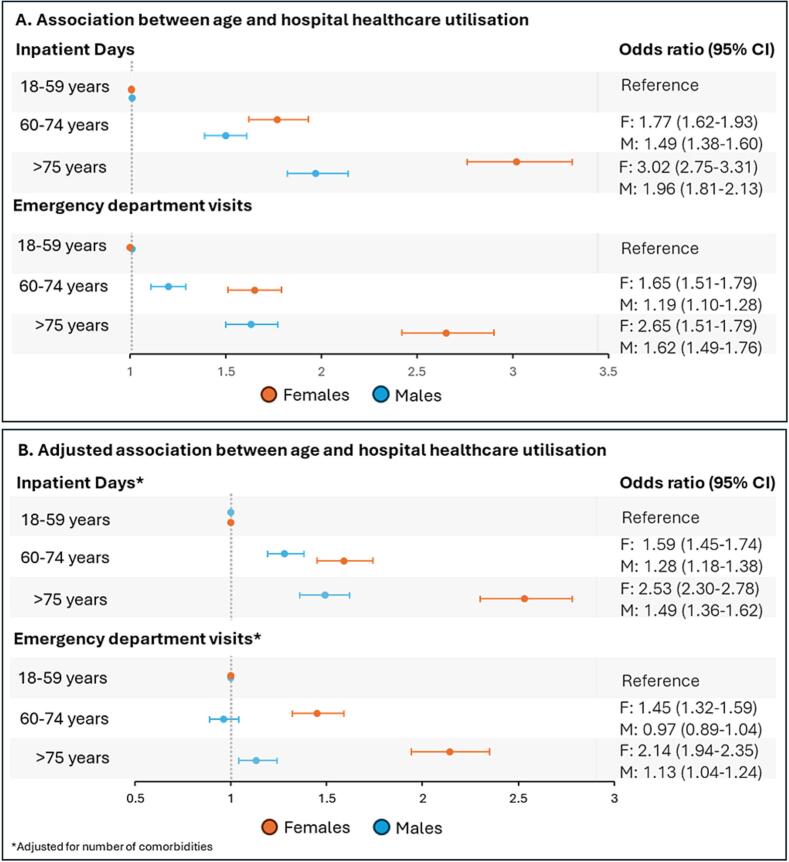
Logistic regression in sexes for the association between age categories and healthcare utilisation. A: Univariate logistic regression. B: Logistic regression adjusted for number of comorbidities.

In univariate logistic regression, both females and males aged 60–74 and those aged ≥ 75 were significantly associated with an increased risk of more inpatient days. Similarly, both females and males aged 60–74 and those aged ≥ 75 were significantly associated with an increased risk of emergency department visits. **(**Fig. 1**A,** Supplemental Table 3) Following adjustment for number of comorbidities both females and males aged 60–74 and those aged ≥ 75 were significantly associated with an increased risk of inpatient days. For females in both age categories and males aged ≥ 75 there was an increased risk of emergency department visits, however there was no association for males aged 60–74 years of age. **(**Fig. 1**B,** Supplemental Table 3).

After adjusting for the interaction between sex and age, females were found to have 1.20 times higher odds of inpatient days compared to males (OR 1.20, 95 % CI: 1.13–1.28, p < 0.001). After adjusting for the interaction between sex and age, females were found to have 1.23 times higher odds of emergency department visits compared to males (OR 1.23, 95 % CI: 1.16–1.31, p < 0.001).

## 4 Discussion

We compared healthcare utilisation between sexes with AF, although females were older and had a higher prevalence of cancer and heart failure compared to males there was no difference in the total number of comorbidities or healthcare utilisation. Age played a role with a higher number of outpatient visits and emergency department visits in younger (18–59 years) females compared to males of similar age. In contrast, in patients aged ≥ 75, males had a higher number of outpatient visits and inpatient days than females of similar age. Logistic regression found an association for inpatient days and emergency department visits for both females and males in the older age categories. Following adjustment for interaction between sex and age, females remained at an increased risk of both inpatient days and emergency visits compared to males. This significant association suggests that, even when accounting for the modifying effect of age, female sex remained at an elevated risk of hospital healthcare utilisation.

Sex differences in AF are well established, with females and males showing distinct patterns in AF presentation, risk profiles, and clinical outcomes [17,18]. Prior data from the ARIC study with AF compared to no AF demonstrated females had higher inpatient and outpatient healthcare utilisation compared to males [19]. More recently, data from Canada demonstrated that despite females having lower prevalence of AF, they had an equal proportion of healthcare utilisation with the exception of males having more emergency department visits [20]. The Canadian data are more in line with our data in which we observed that there was no significant difference between sexes with AF for any healthcare utilisation. However, on further analysis we did observe a difference between sexes based on ages.

A study from the Nationwide Inpatient Sample comparing patients < 65 to those > 65 years of age showed the older patients with AF had longer in hospital stays and were less likely to be discharged home [21]. Interestingly in our cohort, despite females being older than males, when comparing age groups females at a younger age had more outpatient visits compared to younger males, whilst males aged ≥ 75 had more outpatient visits compared to older females. Similarly, younger females saw more medical specialists, whilst for males it was those aged ≥ 75 who saw more specialists. To our knowledge there are no studies that have compared younger and older females and males to determine healthcare utilisation. The results of our study demonstrates that female sex presents with a higher risk of hospital healthcare utilisation than male sex following effect modification for age. This is in line with previous data showing different outcomes between sexes depending on age. Data on young-onset AF patients has shown that, although females present with a markedly different clinical profile compared to males, there is no significant difference in cardiovascular event rates, and AF progression rates are generally comparable [22]. In contrast, findings from the Reappraisal of Atrial Fibrillation: Interaction between HyperCoagulability, Electrical remodelling and Vascular Destabilisation in the Progression of AF (RACE V) study, which involved an older population where women presented at an older age, showed that females had a lower incidence of AF progression compared to males [23].

Importantly, in our cohort it appears that there is a difference in the type of specialist that females see compared to males, females seeing a broader array of specialists compared to males albeit we did not have comprehensive information on the appropriateness or relevance of the specialist or consultations. Females with AF seem to also see cardiologists less than males, irrespective of age. The reasons behind treatment biases in females with AF are not entirely understood, although evidence suggests that sociocultural factors and gender biases in health care delivery may play a significant role [5,24]. Whereas early studies in patients with persistent AF indicated that females treated with rhythm control strategies experienced higher morbidity and mortality, suggesting that a rate control approach might be preferable [25]. The Early Treatment of Atrial Fibrillation for Stroke Prevention (EAST-AFNET 4) Trial underscored the importance of early rhythm control in both sexes, showing equivalent benefits for females and males with no safety differences between them [26]. Further studies to evaluate treatment pathways are needed to identify the different healthcare needs of females and males with AF which may help to further streamline care and referral pathways, preferably according to age and severity and number of underlying comorbidities [27].

### 4.1 Strengths and limitations

This study has several strengths. This study included electronic health records which have all been extracted uniformly from patients from three hospitals in the Netherlands. This indicates that the methodology utilised could be replicated across different hospitals in the Netherlands. In addition, through collection of data through this method the inclusion of typically underrepresented individuals in research enabling more diversity in the population studied [28].

Notwithstanding study strengths we acknowledge several limitations. Firstly, our data rely on electronic health records coded data which therefore did not enable us to distinguish AF specifically from atrial flutter or other supraventricular arrhythmias, or AF type (paroxysmal or persistent) and AF duration (burden). Likewise, the available data were insufficient to calculate CHA_2_DS_2_-VA scores reliably. Whilst most of the arrhythmias were likely due to AF, there was no adjudication of events undertaken. Secondly, comorbidities were likely underestimated, hypertension for example is one of the most prevalent risk factors for AF and only represented 2 % of the patients with AF. This is contrary to what is known with hypertension recognized as one of the most prevalent comorbidities associated with AF affecting an estimated 60 %–80 % of individuals [29]. It is therefore highly probably that hypertension in the present cohort is significantly under reported due to not being a primary diagnosis, potentially resulting in the underrepresentation of multimorbidity. Thirdly, we were only able to access data from 2017 to 2018 for UMCG and Radboud UMC and only had data from 2019 for NWCA therefore were unable to undertake longitudinal analysis. Lastly, due to the nature of the dataset, we lacked access to detailed patient-level clinical information such as prescribed medications, laboratory results, or procedures. This includes no information on specific oral anticoagulant regimens, antiarrhythmic drug use, or catheter ablation procedures. While we had information on the number and type of hospital visits (inpatient and outpatient), we could not assess appropriateness of referrals, procedures undertaken, medication regimens, or adherence. These data are observational so we are unable to exclude residual confounding or establish causal relations.

## 5 Conclusion

When assessing sex differences in this large cohort of patients with AF, there was no difference between sexes in hospital healthcare utilisation. Whilst both males and females with AF had an increase in healthcare utilisation, age played a role. Females remained at an increased risk of both inpatient days and emergency visits compared to males following adjustment for interaction between sex and age, suggesting that female sex remains a high risk of hospital healthcare utilisation.

## CRediT authorship contribution statement

**Melissa E. Middeldorp:** Writing – review & editing, Writing – original draft, Visualization, Validation, Methodology, Investigation, Formal analysis, Conceptualization. **Colinda van Deutekom:** Writing – review & editing. **Liann I. Weil:** Writing – review & editing, Formal analysis, Data curation. **Isabelle C. Van Gelder:** Writing – review & editing, Writing – original draft, Supervision, Methodology, Conceptualization. **Ursula W. De Ruijter:** Writing – review & editing, Validation. **Patrick T. Jeurissen:** Writing – review & editing. **Emelia J. Benjamin:** Writing – review & editing, Validation. **Barbara C. van Munster:** Writing – review & editing, Methodology, Investigation, Data curation, Conceptualization. **Michiel Rienstra:** Writing – review & editing, Writing – original draft, Supervision, Methodology, Formal analysis, Conceptualization.

## Funding

There was no funding for this sudy.

## Declaration of competing interest

The authors declare that they have no known competing financial interests or personal relationships that could have appeared to influence the work reported in this paper.
