# Supplementary material for: Sex differences in hospitalisation and healthcare utilisation for patients with atrial fibrillation Middeldorp et al. Sex differences in healthcare utilisation and AF
Source: Int J Cardiol Heart Vasc. 2025 Jul 12;60:101748. doi: 10.1016/j.ijcha.2025.101748 (PMC12369424; doi:10.1016/j.ijcha.2025.101748)
Supplement: Supplementary Data 1 [file mmc1.docx]

Supplementary Appendix

This appendix has been provided by the authors to give readers additional information about their work.

Supplement to: Middeldorp M, van Deutekom, et al. Sex differences in hospitalisation and healthcare utilisation for patients with atrial fibrillation

**Supplementary Table 1 – List of the 233 diagnosis groups used from the Dutch Hospital Data-Clinical Classification Software (DHD-CCS)**

|  | **Diagnosis group** | **Diagnosis type** |
| --- | --- | --- |
| 1 | Abdominal hernia | Elective |
| 2 | Abdominal pain | Chronic |
| 3 | Acquired foot deformities | Chronic |
| 4 | Acute and chronic tonsillitis | Chronic |
| 5 | Acute and unspecified renal failure | Acute |
| 6 | Acute bronchitis | Acute |
| 7 | Acute cerebrovascular disease | Acute |
| 8 | Acute myocardial infarction | Acute |
| 9 | Adjustment disorders | Chronic |
| 10 | Administrative/social admission | Other |
| 11 | Alcohol-related disorders | Other |
| 12 | Allergic reactions | Chronic |
| 13 | Anal and rectal conditions | Elective |
| 14 | Anxiety disorders | Chronic |
| 15 | Aortic and peripheral arterial embolism or thrombosis | Acute |
| 16 | Aortic; peripheral; and visceral artery aneurysms | Acute |
| 17 | Appendicitis and other appendiceal conditions | Acute |
| 18 | Asthma | Chronic |
| 19 | Bacterial infection; unspecified site | Acute |
| 20 | Benign neoplasm of uterus | Elective |
| 21 | Biliary tract disease | Elective |
| 22 | Birth trauma | Other |
| 23 | Blindness and vision defects | Chronic |
| 24 | Burns | Acute |
| 25 | Calculus of urinary tract | Elective |
| 26 | Cancer of bladder | Oncologic |
| 27 | Cancer of bone and connective tissue | Oncologic |
| 28 | Cancer of brain and nervous system | Oncologic |
| 29 | Cancer of breast | Oncologic |
| 30 | Cancer of bronchus; lung | Oncologic |
| 31 | Cancer of cervix | Oncologic |
| 32 | Cancer of colon | Oncologic |
| 33 | Cancer of esophagus | Oncologic |
| 34 | Cancer of head and neck | Oncologic |
| 35 | Cancer of kidney and renal pelvis | Oncologic |
| 36 | Cancer of liver and intrahepatic bile duct | Oncologic |
| 37 | Cancer of other female genital organs | Oncologic |
| 38 | Cancer of other GI organs; peritoneum | Oncologic |
| 39 | Cancer of other male genital organs | Oncologic |
| 40 | Cancer of other urinary organs | Oncologic |
| 41 | Cancer of ovary | Oncologic |
| 42 | Cancer of pancreas | Oncologic |
| 43 | Cancer of prostate | Oncologic |
| 44 | Cancer of rectum and anus | Oncologic |
| 45 | Cancer of stomach | Oncologic |
| 46 | Cancer of testis | Oncologic |
| 47 | Cancer of thyroid | Oncologic |
| 48 | Cancer of uterus | Oncologic |
| 49 | Cancer; other and unspecified primary | Oncologic |
| 50 | Cancer; other respiratory and intrathoracic | Oncologic |
| 51 | Cardiac and circulatory congenital anomalies | Chronic |
| 52 | Cardiac arrest and ventricular fibrillation | Acute |
| 53 | Cardiac dysrhythmias | Acute |
| 54 | Cataract | Elective |
| 55 | Chronic kidney disease | Chronic |
| 56 | Chronic obstructive pulmonary disease and bronchiectasis | Chronic |
| 57 | Chronic ulcer of skin | Chronic |
| 58 | Coagulation and hemorrhagic disorders | Chronic |
| 59 | Coma; stupor; and brain damage | Acute |
| 60 | Complication of device; implant or graft | Other |
| 61 | Complications of surgical procedures or medical care | Acute |
| 62 | Conditions associated with dizziness or vertigo | Chronic |
| 63 | Conduction disorders | Chronic |
| 64 | Congestive heart failure; non-hypertensive | Chronic |
| 65 | Contraceptive and procreative management | Elective |
| 66 | Coronary atherosclerosis and other heart disease | Chronic |
| 67 | Crushing injury or internal injury | Acute |
| 68 | Cystic fibrosis | Chronic |
| 69 | Deficiency and other anemia | Chronic |
| 70 | Delirium dementia and amnestic and other cognitive disorders | Chronic |
| 71 | Diabetes mellitus with complications | Chronic |
| 72 | Diabetes mellitus without complication | Chronic |
| 73 | Diabetes or abnormal glucose tolerance complicating pregnancy; childbirth; or the puerperium | Chronic |
| 74 | Digestive congenital anomalies | Acute |
| 75 | Diseases of mouth; excluding dental | Elective |
| 76 | Diseases of white blood cells | Chronic |
| 77 | Disorders of lipid metabolism | Chronic |
| 78 | Disorders of teeth and jaw | Other |
| 79 | Disorders usually diagnosed in infancy childhood or adolescence | Chronic |
| 80 | Diverticulosis and diverticulitis | Chronic |
| 81 | Ectopic pregnancy | Other |
| 82 | Encephalitis *(except that caused by tuberculosis or sexually transmitted disease)* | Acute |
| 83 | Endometriosis | Chronic |
| 84 | Epilepsy; convulsions | Chronic |
| 85 | Esophageal disorders | Chronic |
| 86 | Essential hypertension | Chronic |
| 87 | Female infertility | Elective |
| 88 | Fever of unknown origin | Acute |
| 89 | Fluid and electrolyte disorders | Other |
| 90 | Fracture of lower limb | Acute |
| 91 | Fracture of neck of femur *(hip)* | Acute |
| 92 | Fracture of upper limb | Acute |
| 93 | Gastritis and duodenitis | Chronic |
| 94 | Gastroduodenal ulcer *(except hemorrhage)* | Elective |
| 95 | Gastrointestinal hemorrhage | Chronic |
| 96 | Genitourinary congenital anomalies | Chronic |
| 97 | Genitourinary symptoms and ill-defined conditions | Chronic |
| 98 | Glaucoma | Elective |
| 99 | Gout and other crystal arthropathies | Chronic |
| 100 | Headache; including migraine | Chronic |
| 101 | Heart valve disorders | Elective |
| 102 | Hemolytic jaundice and perinatal jaundice | Acute |
| 103 | Hemorrhoids | Elective |
| 104 | Hepatitis | Chronic |
| 105 | HIV infection | Chronic |
| 106 | Hodgkin`s disease | Oncologic |
| 107 | Hyperplasia of prostate | Chronic |
| 108 | Hypertension complicating pregnancy; childbirth and the puerperium | Other |
| 109 | Immunity disorders | Chronic |
| 110 | Immunizations and screening for infectious disease | Acute |
| 111 | Infective arthritis and osteomyelitis *(except that caused by tuberculosis or sexually transmitted disease)* | Chronic |
| 112 | Inflammation; infection of eye *(except that caused by tuberculosis or sexually transmitted disease)* | Acute |
| 113 | Inflammatory conditions of male genital organs | Acute |
| 114 | Inflammatory diseases of female pelvic organs | Acute |
| 115 | Intestinal infection | Acute |
| 116 | Intestinal obstruction without hernia | Acute |
| 117 | Intracranial injury | Acute |
| 118 | Joint disorders and dislocations; trauma-related | Acute |
| 119 | Late effects of cerebrovascular disease | Other |
| 120 | Leukemias | Oncologic |
| 121 | Liver disease, alcohol-related | Chronic |
| 122 | Lung disease due to external agents | Chronic |
| 123 | Lymphadenitis | Chronic |
| 124 | Malaise and fatigue | Chronic |
| 125 | Malignant neoplasm without specification of site | Oncologic |
| 126 | Malposition; malpresentation | Elective |
| 127 | Medical examination/evaluation | Other |
| 128 | Melanomas of skin | Oncologic |
| 129 | Meningitis *(except that caused by tuberculosis or sexually transmitted disease)* | Acute |
| 130 | Menopausal disorders | Chronic |
| 131 | Menstrual disorders | Chronic |
| 132 | Miscellaneous mental health disorders | Chronic |
| 133 | Mood disorders | Chronic |
| 134 | Multiple myeloma | Oncologic |
| 135 | Multiple sclerosis | Chronic |
| 136 | Mycoses | Chronic |
| 137 | Nausea and vomiting | Chronic |
| 138 | Neoplasms of unspecified nature or uncertain behavior | Oncologic |
| 139 | Nephritis; nephrosis; renal sclerosis | Chronic |
| 140 | Nervous system congenital anomalies | Chronic |
| 141 | Non-Hodgkin`s lymphoma | Oncologic |
| 142 | Noninfectious gastroenteritis | Acute |
| 143 | Nonmalignant breast conditions | Elective |
| 144 | Nonspecific chest pain | Acute |
| 145 | Nutritional deficiencies | Chronic |
| 146 | Occlusion or stenosis of precerebral arteries | Other |
| 147 | Open wounds of extremities | Acute |
| 148 | Open wounds of head; neck; and trunk | Acute |
| 149 | Osteoarthritis | Elective |
| 150 | Osteoporosis | Chronic |
| 151 | Other acquired deformities | Elective |
| 152 | Other aftercare | Other |
| 153 | Other and ill-defined cerebrovascular disease | Acute |
| 154 | Other and ill-defined heart disease | Chronic |
| 155 | Other and unspecified benign neoplasm | Elective |
| 156 | Other bone disease and musculoskeletal deformities | Chronic |
| 157 | Other circulatory disease | Chronic |
| 158 | Other CNS infection and poliomyelitis | Chronic |
| 159 | Other complications of birth; puerperium affecting management of mother | Acute |
| 160 | Other complications of pregnancy | Elective |
| 161 | Other congenital anomalies | Chronic |
| 162 | Other connective tissue disease | Chronic |
| 163 | Other diseases of bladder and urethra | Acute |
| 164 | Other diseases of kidney and ureters | Chronic |
| 165 | Other diseases of veins and lymphatics | Chronic |
| 166 | Other disorders of stomach and duodenum | Chronic |
| 167 | Other ear and sense organ disorders | Chronic |
| 168 | Other endocrine disorders | Elective |
| 169 | Other eye disorders | Elective |
| 170 | Other female genital disorders | Chronic |
| 171 | Other fractures | Acute |
| 172 | Other gastrointestinal disorders | Chronic |
| 173 | Other hematologic conditions | Chronic |
| 174 | Other hereditary and degenerative nervous system conditions | Chronic |
| 175 | Other infections; including parasitic | Acute |
| 176 | Other inflammatory condition of skin | Chronic |
| 177 | Other injuries and conditions due to external causes | Acute |
| 178 | Other liver diseases | Chronic |
| 179 | Other lower respiratory disease | Chronic |
| 180 | Other male genital disorders | Elective |
| 181 | Other nervous system disorders | Chronic |
| 182 | Other non-epithelial cancer of skin | Oncologic |
| 183 | Other non-traumatic joint disorders | Chronic |
| 184 | Other nutritional; endocrine; and metabolic disorders | Chronic |
| 185 | Other perinatal conditions | Elective |
| 186 | Other pregnancy and delivery including normal | Elective |
| 187 | Other screening for suspected conditions *(not mental disorders or infectious disease)* | Elective |
| 188 | Other skin disorders | Chronic |
| 189 | Other upper respiratory disease | Chronic |
| 190 | Other upper respiratory infections | Elective |
| 191 | Otitis media and related conditions | Acute |
| 192 | Pancreatic disorders *(not diabetes)* | Acute |
| 193 | Paralysis | Chronic |
| 194 | Parkinson`s disease | Chronic |
| 195 | Pathological fracture | Chronic |
| 196 | Peri-; endo-; and myocarditis; cardiomyopathy *(except that caused by tuberculosis or sexually transmitted disease)* | Chronic |
| 197 | Peripheral and visceral atherosclerosis | Chronic |
| 198 | Peritonitis and intestinal abscess | Acute |
| 199 | Phlebitis; thrombophlebitis and thromboembolism | Acute |
| 200 | Pleurisy; pneumothorax; pulmonary collapse | Acute |
| 201 | Pneumonia *(except that caused by tuberculosis or sexually transmitted disease)* | Acute |
| 202 | Poisoning by nonmedicinal substances | Acute |
| 203 | Previous C-section | Other |
| 204 | Prolapse of female genital organs | Chronic |
| 205 | Pulmonary heart disease | Chronic |
| 206 | Regional enteritis and ulcerative colitis | Chronic |
| 207 | Rehabilitation care; fitting of prostheses; and adjustment of devices | Other |
| 208 | Residual codes; unclassified | Chronic |
| 209 | Respiratory distress syndrome | Other |
| 210 | Respiratory failure; insufficiency; arrest *(adult)* | Acute |
| 211 | Retinal detachments; defects; vascular occlusion; and retinopathy | Elective |
| 212 | Rheumatoid arthritis and related disease | Chronic |
| 213 | Schizophrenia and other psychotic disorders | Chronic |
| 214 | Screening and history of mental health and substance abuse codes | Other |
| 215 | Secondary malignancies | Oncologic |
| 216 | Septicemia *(except in labor)* | Acute |
| 217 | Sexually transmitted infections *(not HIV or hepatitis)* | Acute |
| 218 | Sickle cell anemia | Chronic |
| 219 | Skin and subcutaneous tissue infections | Acute |
| 220 | Skull and face fractures | Acute |
| 221 | Spinal cord injury | Acute |
| 222 | Spondylosis; intervertebral disc disorders; other back problems | Chronic |
| 223 | Sprains and strains | Acute |
| 224 | Substance-related disorders | Chronic |
| 225 | Superficial injury; contusion | Acute |
| 226 | Syncope | Acute |
| 227 | Systemic lupus erythematosus and connective tissue disorders | Chronic |
| 228 | Thyroid disorders | Chronic |
| 229 | Transient cerebral ischemia | Acute |
| 230 | Tuberculosis | Chronic |
| 231 | Urinary tract infections | Elective |
| 232 | Varicose veins of lower extremity | Elective |
| 233 | Viral infection | Acute |

**Supplement table 2: Type of medical specialist seen by females and males by age category**

|  | **Female** | | | |  | **Male** | | | |
| --- | --- | --- | --- | --- | --- | --- | --- | --- | --- |
| **Years of age:** | **18-59** | **60-74** | ≥**75** | **p-value** |  | **18-59** | **60-74** | ≥**75** | **p-value** |
| **N=** | 473 | 981 | 820 |  |  | 634 | 1,426 | 793 |  |
| **Medical Specialist** |  |  |  |  |  |  |  |  |  |
| Anaesthesiology, n (%) | 10 (2.1) | 27 (2.8) | 29 (3.5) | 0.32 |  | 12 (1.9) | 20 (1) | 23 (3) | 0.048 |
| Cardiology, n (%) | 290 (61.3) | 811 (82.7) | 704 (85.9) | <0.001 |  | 576 (90.9) | 1325 (93) | 721 (91) | 0.14 |
| Cardiothoracic surgery, n (%) | 8 (1.7) | 11 (1.1) | 4 (0.5) | 0.37 |  | 30 (4.7) | 29 (2) | 5 (1) | 0.001 |
| Clinical genetics, n (%) | 12 (2.5) | 8 (0.8) | 2 (0.2) | 0.004 |  | 9 (1.4) | 10 (1) | 0 | 0.04 |
| Dermatology, n (%) | 20 (4.2) | 85 (8.7) | 111 (13.5) | <0.001 |  | 35 (5.5) | 125 (9) | 126 (16) | 0.016 |
| Gastroenterology, n (%) | 65 (13.7) | 93 (9.5) | 97 (11.4) | 0.043 |  | 44 (6.9) | 119 (8) | 88 (11) | <0.001 |
| General surgery, n (%) | 55 (11.6) | 146 (14.9) | 154 (18.8) | 0.002 |  | 59 (9.3) | 214 (15) | 168 (21) | <0.001 |
| Geriatrics, n (%) | 3 (1) | 15 (2) | 75 (9) | <0.001 |  | 3 (1) | 30 (2) | 72 (9) | <0.001 |
| Gynaecology, n (%) | 76 (16) | 66 (7) | 78 (10) | <0.001 |  | 1 (0.2) | 0 | 0 | 0.17 |
| Internal medicine, n (%) | 150 (32) | 268 (27) | 249 (30) | 0.16 |  | 128 (20) | 378 (27) | 304 (38) | <0.001 |
| Neurology, n (%) | 65 (14) | 137 (14) | 147 (18) | 0.037 |  | 78 (12) | 237 (17) | 167 (21) | <0.001 |
| Neurosurgery, n (%) | 8 (2) | 19 (2) | 18 (2) | 0.82 |  | 5 (1) | 24 (2) | 15 (2) | 0.20 |
| Ophthalmology, n (%) | 29 (6) | 77 (8) | 105 (13) | <0.001 |  | 28 (4) | 120 (8) | 103 (12) | <0.001 |
| Orthopaedic surgery, n (%) | 25 (5) | 88 (9) | 111 (14) | <0.001 |  | 35 (6) | 89 (6) | 84 (11) | <0.001 |
| Otorhinolaryngology, n (%) | 27 (6) | 61 (6) | 72 (9) | 0.05 |  | 21 (3) | 111 (8) | 90 (11) | <0.001 |
| Physiatry Rehabilitation, n (%) | 3 (1) | 6 (1) | 3 (0.4) | 0.73 |  | 6 (1) | 19 (1) | 4 (1) | 0.17 |
| Plastic surgery, n (%) | 18 (4) | 16 (2) | 16 (2) | 0.025 |  | 6 (1) | 32 (2) | 17 (2) | 0.12 |
| Psychiatry, n (%) | 1 (0.2) | 1 (0.1) | 0 | 0.58 |  | 1 (0.2) | 3 (0.2) | 1 (0.1) | 0.94 |
| Pulmonology, n (%) | 40 (9) | 134 (14) | 123 (15) | 0.003 |  | 57 (9) | 237 (17) | 149 (19) | <0.001 |
| Radiotherapy, n (%) | 97 (21) | 137 (14) | 85 (10) | <0.001 |  | 11 (2) | 76 (5) | 57 (7) | <0.001 |
| Rheumatology, n (%) | 29 (6) | 71 (7) | 72 (9) | 0.20 |  | 26 (4) | 81 (6) | 49 (6) | 0.20 |
| Urology, n (%) | 14 (3) | 48 (5) | 35 (4) | 0.23 |  | 27 (4) | 181 (13) | 170 (21) | <0.001 |

**Supplement table 3: Linear regression in sexes for the association between age categories and healthcare utilisation.**

| **Inpatient Visits** | **Coefficients (95% CI)** | **p-Value** |
| --- | --- | --- |
| 18-59 years | Reference |  |
| 60-74 years | F: 1.45 (1.12-1.78)  M: 1.21 (0.89-1.53) | <0.001  <0.001 |
| >75 years | F: 2.68 (2.33-3.03)  M: 2.23 (1.87-2.59) | <0.001  <0.001 |
| **ED visits** |  |  |
| 18-59 years | Reference |  |
| 60-74 years | F: 0.18 (0.13-0.22)  M: 0.92 (0.05-0.13) | <0.001  <0.001 |
| >75 years | F: 0.48 (0.43-0.53)  M: 0.28 (0.23-0.33) | <0.001  <0.001 |
